# Successful Ultrasound-Guided Femoral Nerve Blockade and Catheterization in a Patient with Von Willebrand Disease

**DOI:** 10.1155/2015/106306

**Published:** 2015-05-31

**Authors:** Youmna E. DiStefano, Michael D. Lazar

**Affiliations:** Department of Anesthesiology, New York Medical College, Valhalla, NY 10595, USA

## Abstract

Peripheral nerve blockade (PNB) is superior to neuraxial anesthesia and/or opioid therapy for perioperative analgesia in total knee replacement (TKR). Evidence on the safety of PNB in patients with coagulopathy is lacking. We describe the first documented account of continuous femoral PNB for perioperative analgesia in a patient with Von Willebrand Disease (vWD). Given her history of opioid tolerance and after an informative discussion, a continuous femoral PNB was planned for in this 34-year-old female undergoing TKR. A Humate-P intravenous infusion was started and the patient was positioned supinely. Using sterile technique with ultrasound guidance, a Contiplex 18 Gauge Tuohy needle was advanced in plane through the fascia iliaca towards the femoral nerve. A nerve catheter was threaded through the needle and secured without complications. Postoperatively, a levobupivacaine femoral catheter infusion was maintained, and twice daily Humate-P intravenous infusions were administered for 48 hours; enoxaparin thromboprophylaxis was initiated thereafter. The patient was discharged uneventfully on postoperative day 4. Given documentation of delayed, unheralded bleeding from PNB in coagulopathic patients, we recommend individualized PNB in vWD patients. Multidisciplinary team involvement is required to guide factor supplementation and thromboprophylaxis, as is close follow-up to elicit signs of bleeding throughout the delayed postoperative period.

## 1. Introduction

Von Willebrand disease (vWD) is the most common inherited bleeding disorder. Despite advances in the molecular diagnosis and treatment of this disease, the literature remains scarce as to the perioperative management of vWD patients undergoing orthopedic and other types of surgery. In particular, joint replacement surgery is amenable to various regional anesthetic techniques, including neuraxial anesthesia, lumbar plexus blockade, and peripheral nerve blockade (PNB). Evidence has established the superiority of PNB to neuraxial anesthesia and/or opioid therapy in total knee replacement (TKR) patients [1–3] and in orthopedic surgery patients on thromboprophylaxis (ppx) [4]. While PNB among other modalities has been associated with superior pain scores and a more favorable side effect profile, its use in patients with inherited or acquired coagulopathy may carry potentially catastrophic hemorrhagic complications. Certainly, the available evidence regarding the safety of PNB in coagulopathic patients is insufficient to date. To our knowledge, this case study is the first documented account of a continuous femoral nerve catheter insertion for intra- and postoperative pain control in a patient with Von Willebrand disease.

## 2. Case Report

The patient presented has granted us permission to publish this report. She is a 34-year-old female diagnosed with type 1 vWD associated with a hypercoagulable state, as evidenced by superficial thrombophlebitis and 3 spontaneous abortions in the past. She had a long history of debilitating left knee chondromalacia patellae secondary to a slip and fall injury at age 15; this progressed to advanced patellofemoral arthrosis, resulting in significantly decreased ambulatory capacity and constant knee pain refractory to large doses of nonsteroidal anti-inflammatory medications and Percocet. The patient had failed physical therapy, multiple intra-articular steroid injections, multiple arthroscopic debridements, and tibial tubercle osteotomy with lateral retinacular release; she was thus scheduled to undergo a left TKR. Her medical history was otherwise significant for mild intermittent asthma, and her surgical history was notable for inferior vena cava filter placement, left wrist ganglion cyst excision, 3 dilatation and curettage procedures, and a Caesarean section complicated by severe intraoperative hemorrhage requiring massive transfusion with packed red blood cells, fresh frozen plasma, and factor VIII concentrate. Her height and weight were 170 cm and 70 kg, respectively. She was treated with Singulair, albuterol, nonsteroidal anti-inflammatory medications, and Percocet; she also had an allergy to penicillin.

During the preanesthetic evaluation, the patient expressed an interest in a femoral nerve block for postoperative pain control, given her history of opioid tolerance and in light of a self-review of the pertinent literature. The risks and benefits of PNB and general endotracheal anesthesia (GETA) were discussed with the patient, with particular reference to her coagulopathy and the risk of bleeding complications from regional anesthesia. The anesthetic plan consisted of intravenous (IV) infusion of Humate-P in compliance with the hematologist's recommendations and it was dosed using body weight and baseline plasma vWF ristocetin cofactor activity. This was to be followed by ultrasound-guided continuous left femoral nerve blockade and GETA.

In the operating room, the patient was positioned supinely and standard American Society of Anesthesiologists monitors were applied. The patient was premedicated with midazolam 2 mg IV and fentanyl 50 mcg IV; the block site was prepared with chlorhexidine and draped in a sterile fashion. An IV infusion of 1876 units of Humate-P was initiated prior to the start of the block. With the ultrasound transducer sterilely covered and positioned transversely over the left inguinal crease, the femoral artery and femoral nerve were identified. Using a Contiplex 18 Gauge Tuohy continuous nerve block tray, the site was locally infiltrated with 6 mL of 2% lidocaine with 1 : 100,000 epinephrine. The needle was positioned in plane and advanced medially toward the femoral nerve. Once adequate local anesthetic spread was visualized, the nerve catheter was threaded through the needle and advanced 4 cm past the needle tip. A total of 20 mL of 0.5% levobupivacaine and 10 mL of 2% lidocaine with 1 : 100,000 epinephrine was injected during the procedure. The catheter was secured at the skin with a Tegaderm dressing and reinforced with adhesive tape. There were no signs of vascular trauma, nerve injury, or bleeding throughout the block procedure.

This was followed by standard induction of general anesthesia, endotracheal intubation, and maintenance with inhalational anesthetic.

Intraoperatively, a left lower extremity tourniquet was applied at a pressure of 250 psi for 76 minutes. Towards the end of the operation, 10 mL of 0.5% levobupivacaine was injected through the femoral catheter. Total surgical time was 79 minutes and blood loss was approximately 50 mL. The patient emerged from anesthesia and was extubated uneventfully. She made no complaints and was transferred to the postanesthesia care unit, where a femoral catheter infusion of 0.2% levobupivacaine at 12 mL per hour was started and maintained for 2 days postoperatively. This was supplemented through postoperative day (POD) 1 with hydromorphone patient-controlled analgesia; the patient required a total of 7.6 mg of hydromorphone during the 24-hour succeeding surgery. As per recommendations from the consulting hematology team, the patient also received 1600 units of Humate-P every 12 hours for 48 hours postoperatively. After the 4th and last dose of Humate-P was administered (i.e., on the night of POD 2), ppx was initiated with enoxaparin 30 mg subcutaneously daily. The femoral nerve catheter was removed intact on POD 2. The patient's postoperative course was uncomplicated and she was discharged from the hospital on POD 4.

## 3. Discussion

The need for orthopedic surgery among vWD patients is not uncommon, especially considering the risk of development of disabling arthropathy from repeated bleeding into the involved joints. Our patient was suffering from patellofemoral arthrosis unrelated to her coagulopathy. At any rate, surgical intervention in vWD patients is missing evidence-based standards for safe, quality perioperative management.

According to a meta-analysis of 504 TKRs, PNB is associated with equivalent analgesia, significantly lower rates of hypotension and urinary retention, and higher patient satisfaction as compared to neuraxial blocks [2]. Moreover, while epidural and spinal hematomas carry an alarmingly high risk of permanent neurologic disability, hematomas resulting from PNB tend to develop in more compliant spaces and are theoretically less likely to cause catastrophic nerve impingement [5]. This consideration, however, is by no means definitive and certainly cannot be assumed in states of inherited or acquired coagulopathy as in vWD cases.

Table 1 summarizes the available literature on the outcomes of femoral nerve blockade in patients on ppx and/or at above-standard bleeding risk [6–11]. Two case reports [10, 11] involved temporary lower extremity motor impairment, and one prospective series [9] detailed a case of retroperitoneal hematoma associated with permanent quadriceps femoris denervation. The concerned patient was reportedly self-administering 1 g of aspirin daily pre- and postoperatively, unbeknownst to her caregivers [9]. Importantly, the signs of PNB-associated bleeding described in the literature (Table 1) were not apparent until later in the postoperative period, between POD 2 [9] and POD 10 [11]. It also remains unclear whether or not the bleeding was associated with initiation of ppx therapy versus vascular puncture or delayed intravascular migration of femoral nerve catheters. The unheralded, delayed presentation of PNB-related hemorrhage could account for the higher rate of neurologic complications as a result of the insidious accumulation of blood at the blockade site; this is in contrast to such procedures as transfemoral cardiac catheterization, which, although performed on anticoagulated patients by definition, are marked by a higher incidence of acute blood loss and vascular complications. Lower postprocedure vigilance and surveillance for bleeding could also be incriminated in the insidious yet catastrophic neurologic outcomes of PNB-related hemorrhage, which is in stark contrast to the close patient observation and femoral compression performed immediately after cardiac catheterization.

Perioperative supplementation of vWD patients with Von Willebrand factor/factor VIII (vWF/FVIII) concentrates is commonly recommended by hematologists to mitigate the risk of massive or uncontrolled bleeding. Humate-P is a human plasma-derived vWF/FVIII concentrate with an extensive, three-decade track record of no thrombosis and no cases of viral transmission [12, 13]. It is recommended for surgical prophylaxis and spontaneous bleeding treatment in all types of vWD and in vWD cases that are refractory to desmopressin. As per recommendations from the patient's hematologist, she received a Humate-P preoperative dose of 1876 units, followed by postoperative maintenance doses of 1600 units every 12 hours. These correspond to Humate-P surgical prophylaxis doses, which are calculated based on a formula integrating body weight and baseline plasma vWF ristocetin cofactor activity [14].

The 1st dose of prophylactic enoxaparin was administered to our patient after 48 hours of supplementation with Humate-P, at which point it was felt by the hematology team that the combination of postoperative time and loaded Humate-P dose was favorable for adequate hemostasis. According to the American Academy of Orthopaedic Surgeons (AAOP) guidelines, ppx after total hip or knee replacement procedures in patients at elevated risk for both pulmonary embolism and major bleeding (as in our case subject) should consist of aspirin 325 mg twice daily, or warfarin (with a goal international normalized ratio (INR) less than or equal to 2.0), or none, in addition to the use of mechanical prophylaxis [15]. The latest ASRA consensus report, however, acknowledges important shortcomings in the current literature on ppx indications. These include failure to ensure adequate case mix balance between patients at risk of thrombosis and patients at risk for major bleeding, the deliberate exclusion of patients with preexisting coagulopathy, and the use of surrogate endpoints instead of the prescribed primary outcomes (i.e., symptomatic deep venous thrombosis and pulmonary embolism) [5].

Until authoritative evidence is generated to guide perioperative anesthetic care in patients with bleeding disorders, we recommend that vWD patients engage in an informed and educated decision regarding their plan of analgesia. Regional anesthetic techniques must be considered on a case-by-case basis, with careful weighing of the inherent risks and benefits. Multidisciplinary involvement by experts in hematology, surgery, anesthesiology, and nursing is required as is adherence to timely clotting factor supplementation and appropriate ppx. Standard PNB techniques using advanced technology, such as ultrasound guidance, are likely advantageous. Additionally, PNB sites and catheters must be carefully inspected and symptoms elicited for signs of bleeding throughout the postoperative period extending several days after surgery.

## Figures and Tables

**Table 1:** Available literature on outcomes of femoral nerve blockade in patients on thromboprophylaxis and/or at above-standard bleeding risk.

Author	Study type	Nb. of blocks	Type of block	ppx or bleeding risk	Complication(s) from femoral block
Chelly and Schilling [6]	Retrospective cohort	1790	Continuous femoral	10% aspirin ppx 20% LMWH ppx 10% fondaparinux ppx 60% warfarin ppx	0

Vanarase et al. [7]	Retrospective cohort	21	Single-shot femoral or sciatic or both	2 vWD patients 13 hemophilia patients	0

Sripada et al. [8]	Case report	2	Single-shot femoral and sciatic	2 hemophilia patients	0

Wiegel et al. [9]	Retrospective cohort	628	Continuous femoral	Not specified	36 vascular punctures 1 retroperitoneal hematoma causing permanent nerve injury^*∗*^

Bickler et al. [10]	Case report	2	Continuous femoral and sciatic	LMWH ppx	1 swelling & discoloration at block site with temporary lower extremity motor impairment 1 delayed oozing at catheter site

Rodríguez et al. [11]	Case report	1	Single-shot femoral and sciatic	Factor XI deficiency, LMWH ppx	Perineural hematoma with temporary lower extremity paralysis

^*∗*^Nerve injury was evidenced by complete quadriceps femoris denervation. The concerned patient was reportedly self-administering 1 g of aspirin daily pre- and postoperatively, unbeknownst to her caregivers.

Nb. indicates number; ppx, thromboprophylaxis; LMWH, low molecular weight heparin.
